# Age- and sex-specific trends in dementia mortality among people with and without diabetes: a multi-country population-based analysis

**DOI:** 10.1007/s00125-026-06725-2

**Published:** 2026-04-21

**Authors:** Kanika Mehta, Jedidiah I. Morton, Lei Chen, Kaarin J. Anstey, Bendix Carstensen, Edward W. Gregg, Martti Arffman, Gillian L. Booth, Luan Manh Chu, Kelly Fleetwood, Archana Singh-Manoux, Sandrine Fosse-Edorh, Marie Guion, Padma Kaul, Calvin Ke, Ilmo Keskimäki, Susanne Boel Graversen, Tinne Laurberg, Henrik Støvring, Sarah H. Wild, Jonathan E. Shaw, Dianna J. Magliano

**Affiliations:** 1 Baker Heart and Diabetes Institute, Melbourne, VIC Australia; 2 Faculty of Medicine, Dentistry and Health Sciences, University of Melbourne, Melbourne, VIC Australia; 3 School of Public Health and Preventive Medicine, Monash University, Melbourne, VIC Australia; 4 Centre for Medicine Use and Safety, Monash University, Melbourne, VIC Australia; 5 School of Psychology, University of New South Wales, Sydney, NSW Australia; 6 Neuroscience Research Australia, Sydney, NSW Australia; 7 UNSW Ageing Futures Institute, University of New South Wales, Sydney, NSW Australia; 8 Clinical Epidemiology, Steno Diabetes Centre Copenhagen, Herlev, Denmark; 9 School of Population Health, Royal College of Surgeons in Ireland, University of Medicine and Health Sciences, Dublin, Ireland; 10 Department of Epidemiology and Biostatistics, School of Public Health, Imperial College London, London, UK; 11 Department of Healthcare and Social Welfare, Finnish Institute for Health and Welfare, Helsinki, Finland; 12 Department of Medicine, University of Toronto, Toronto, ON Canada; 13 Institute of Health Policy, Management and Evaluation, University of Toronto, Toronto, ON Canada; 14 ICES, Toronto, ON Canada; 15 Provincial Research Data Services, Alberta Health Services, Edmonton, AB Canada; 16 Alberta SPOR SUPPORT Unit, Data and Research Services, Alberta Health Services, Edmonton, AB Canada; 17 Usher Institute, University of Edinburgh, Edinburgh, UK; 18 Université Paris Cité, Inserm U1153, Epidemiology of Ageing and Neurodegenerative Diseases, Paris, France; 19 Faculty of Brain Sciences, University College London, London, UK; 20 Department of Non-Communicable Diseases and Trauma, Santé Publique France, Saint-Maurice, France; 21 Department of Medicine, Faculty of Medicine and Dentistry, University of Alberta, Edmonton, AB Canada; 22 Canadian VIGOUR Center, Edmonton, AB Canada; 23 Alberta Diabetes Institute, University of Alberta, Edmonton, AB Canada; 24 Department of Medicine, Toronto General Hospital, University Health Network, Toronto, ON Canada; 25 Steno Diabetes Center Aarhus, Aarhus University Hospital, Aarhus N, Denmark; 26 Department of Pathology, Aarhus University Hospital, Aarhus N, Denmark; 27 Department of Biomedicine, Aarhus University, Aarhus, Denmark; 28 School of Life Sciences, La Trobe University, Melbourne, VIC Australia

**Keywords:** Administrative datasets, Dementia, Mortality trends, Multi-country

## Abstract

**Aims/hypothesis:**

Large-scale data on age- and sex-specific dementia mortality trends among people with diabetes remain limited, as most previous studies have been restricted to single countries or have not distinguished mortality by diabetes status. We estimated age- and sex-specific time trends in dementia mortality among individuals with and without diabetes from high-income jurisdictions.

**Methods:**

We analysed aggregated mortality and demographic data using registries and administrative sources in Australia, Canada (Alberta and Ontario), France, Denmark, Finland and Scotland from 2000 to 2023. Poisson regression was used to estimate mortality rates for dementia as the underlying cause of death in people with and without diabetes at 60, 70, 80 and 90 years of age.

**Results:**

A total of 114,559 and 589,706 dementia deaths were identified in over 42 and 244 million person-years of follow-up for individuals with and without diagnosed diabetes, respectively. Dementia mortality trends varied by age and jurisdiction but were generally consistent for both sexes. At younger ages (e.g. 60 and 70 years), the dementia mortality trends did not suggest any meaningful increases or decreases, except for in Scotland, which reported increasing dementia mortality over time only for those with diabetes. At older ages (e.g. 80 and 90 years), however, increases in dementia mortality were observed in most jurisdictions, ranging from 7.6% to 42.4% per 5 years. The magnitude of the increases was generally greater for those with diabetes. Mortality from dementia subtypes (e.g. Alzheimer’s disease and vascular dementia) also increased over time in individuals aged 40–89 years, with greater increases in mortality rates for individuals with diabetes, specifically in Australia and Scotland.

**Conclusions/interpretation:**

Increases in dementia mortality were observed for those aged 80 years and above and were most marked for people with diabetes. These findings highlight the growing burden of dementia for health systems.

**Graphical Abstract:**

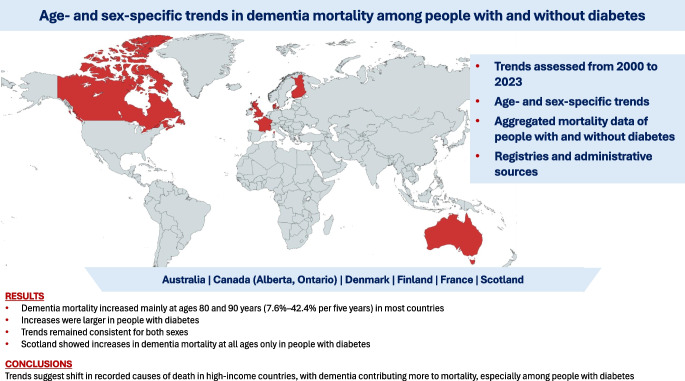

**Supplementary Information:**

The online version of this article (10.1007/s00125-026-06725-2) contains peer-reviewed but unedited supplementary material.


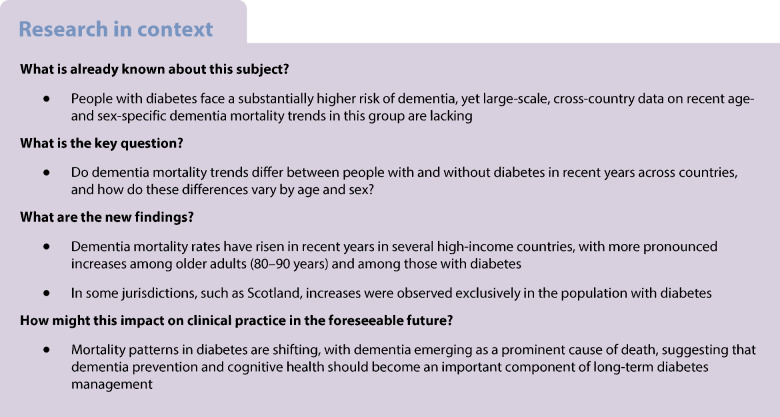


## Introduction

Ongoing advances in diabetes management and healthcare have contributed to a marked reduction in mortality among people with diabetes in high-income countries [1, 2]. Due to this encouraging development, the landscape of diabetes-related complications continues to evolve, and we are now witnessing the emergence of ‘non-traditional’ complications of diabetes such as dementia, cancer and liver disease [3].

Diabetes is widely recognised as being linked to higher risk of dementia [4], and the diversification of diabetes complications raises critical questions regarding the potential impact on mortality from dementia among individuals with diabetes. Since the early 2000s, the proportion of deaths attributed to dementia has risen in people with diabetes, while deaths from traditional vascular causes have declined [5, 6]. A recent large-scale study, comprising record-linkage data for approximately two million Australians, reported people with type 2 diabetes to have higher risk of dementia-related death relative to those without diabetes [7].

A rise in diabetes-associated dementia mortality is also apparent in other high-income countries [6, 8, 9], with some reporting an increasing gap in dementia mortality between individuals with and without diabetes in recent years [6]. Whether age and sex impact diabetes-associated dementia mortality remains unclear. For instance, higher blood glucose levels have been previously associated with higher risk of late-onset dementia (affects people aged >65 years) but not early-onset dementia (affects people aged <65 years), suggesting differences in aetiology of early- and late-onset dementia [10]. There could also be sex-specific differences in dementia mortality, as dementia mortality is generally higher among women, while men with diabetes may experience more severe vascular complications [11, 12]. Further, long-term trends of excess dementia mortality (in people with vs without diabetes) are inadequately described, necessitating further research.

Accordingly, we examined age- and sex-specific trends in dementia mortality between 2000 and 2023 using record-linkage data from seven nationally or regionally representative administrative datasets of people with and without diabetes. We further examined age- and sex-specific trends in mortality for dementia subtypes, namely Alzheimer’s disease, vascular dementia and unspecified dementia.

## Methods

### Data sources

The present study analysed aggregated data from seven administrative databases from an international diabetes consortium [13]. The consortium compiled aggregated data on the causes of mortality from more than 20 national or regional administrative data sources around the world [13]. The initial eligibility criteria for inclusion have been described previously [13]. For the present study, the data sources were required to meet the following criteria: (1) have ongoing enrolment of new diabetes cases (or regular recruitment of new independent cohorts); (2) record cause-specific death counts for individuals with and without diabetes; and (3) provide age- and sex-specific data. We collated aggregated data from seven jurisdictions (Australia, Alberta in Canada, Ontario in Canada, Denmark, Finland, France and Scotland). Data collection items included population size, prevalent diabetes counts, dementia death counts and person-years of follow-up for individuals with and without diabetes, stratified by sex and 5 year age groups at death (<40, 40–44, 45–49, …, 85–89, >90 years) for each calendar year from 2000 onwards (or the earliest available year after 2000) (Table 1). Sex was recorded in the administrative or vital statistics records and provided as a binary variable (male or female). All included jurisdictions were from high-income countries with established healthcare systems and comprehensive population-based health registries.

**Table 1:** Summary characteristics of the included populations

Jurisdiction	Data type	Years analysed	Diabetes status	Sex	No. of dementia deaths at age 60–69 years	PY at age 60–69 years	No. of dementia deaths at age 70–79 years	PY at age 70–79 years	No. of dementia deaths at age 80–89 years	PY at age 80–89 years	No. of dementia deaths at age 90+ years	PY at age 90+ years
Australia	Registry	2005–2021	Diabetes	Female	125	1,499,883	1405	1,401,185	5545	798,161	3905	131,574
			Diabetes	Male	243	1,995,437	1681	1,693,450	4639	730,048	1983	77,153
			No diabetes	Female	1157	14,144,966	7105	8,895,864	34,723	4,986,432	40,168	1,306,952
			No diabetes	Male	1142	13,140,962	6316	7,804,436	20,784	3,441,717	12,909	576,438
Canada (Alberta)	Administrative	2005–2020	Diabetes	Female	70	428,484	392	338,322	1670	200,930	1601	47,461
			Diabetes	Male	93	580,396	525	413,557	1488	181,989	720	25,757
			No diabetes	Female	167	2,484,426	1128	1,328,041	5123	717,214	6839	204,787
			No diabetes	Male	159	2,357,699	983	1,113,600	3226	470,707	2245	82,397
Canada (Ontario)	Administrative	2013–2018	Diabetes	Female	87	778,008	682	644,796	2800	382,941	2487	81,466
			Diabetes	Male	117	960,194	808	733,708	2320	334,743	1067	43,731
			No diabetes	Female	350	4,220,845	1895	2,409,267	9391	1,360,795	12,009	440,814
			No diabetes	Male	318	3,764,718	1600	1,974,349	5506	925,194	3726	193,259
Denmark	Registry	2002–2019	Diabetes	Female	67	420,803	482	418,000	1809	242,674	1231	46,408
			Diabetes	Male	88	611,725	589	493,163	1320	182,494	457	18,711
			No diabetes	Female	562	5,464,917	3777	3,638,898	15,023	1,927,108	13,466	473,406
			No diabetes	Male	553	5,109,423	2953	3,000,267	8146	1,163,360	3789	161,995
Finland	Registry	2000–2023	Diabetes	Female	191	1,040,778	2566	1,170,464	13,074	780,889	10,724	148,366
			Diabetes	Male	332	1,365,738	2906	1,145,163	7931	449,774	3000	45,694
			No diabetes	Female	1118	6,976,974	8747	4,918,377	40,228	2,632,852	40,244	563,676
			No diabetes	Male	1142	6,104,957	7340	3,601,827	21,099	1,347,349	10,653	172,058
France	Administrative	2013–2020	Diabetes	Female	154	3,036,384	1396	2,781,471	6633	1,945,500	4210	333,702
			Diabetes	Male	249	4,142,434	1703	3,273,030	5166	1,535,011	1719	152,187
			No diabetes	Female	1565	27,515,046	9982	17,362,823	62,488	12,213,623	67,204	3,531,569
			No diabetes	Male	1595	22,249,149	8231	13,010,125	31,951	6,802,183	17,464	1,187,712
Scotland	Registry	2006–2020	Diabetes	Female	76	441,445	901	464,769	3213	259,016	1767	40,303
			Diabetes	Male	121	636,755	986	535,327	2329	214,315	716	20,810
			No diabetes	Female	629	4,287,915	4536	3,015,543	8151	562,997	7536	131,244
			No diabetes	Male	579	3,787,145	3381	2,332,628	4313	354,150	2292	54,804

PY, person-years of follow-up

### Ascertainment of diabetes status

Diabetes definitions for all data sources are presented in the electronic supplementary material (ESM) Table 1. Specifically, for Australia and Scotland, diabetes was defined based on clinical diagnosis by healthcare professionals and according to the ICD-10 codes, respectively. For Canada (Alberta and Ontario), diabetes was defined using an algorithm incorporating one or more hospitalisations or two or more physician claims, within a 2 year period, with evidence of diabetes. For Denmark, Finland and France, algorithms, which included clinical diagnosis, linkage to medication/reimbursement registries and measurement of blood glucose/HbA_1c_, were used to define diabetes.

### Ascertainment of dementia mortality

Dementia death was ascertained via linkage to each jurisdiction’s death registries. Death from dementia was defined if any of the dementia ICD-10 (G30, F01 and F03) codes were reported as the underlying cause of death. Deaths were classified according to dementia subtypes based on specific ICD-10 codes when available (Australia, Denmark, Finland and Scotland). Dementia subtypes were defined by the ICD-10 codes G30 for Alzheimer’s disease, F01 for vascular dementia and F03 for unspecified dementia.

### Quality of included data

The risk of bias was assessed independently by two authors (DJM and LC) using a modified Newcastle–Ottawa Scale (refer to ESM Methods). A score of 0–5, 6–7 and 8–9 suggested a high, medium and low risk of bias, respectively. Disagreements were resolved through discussion with a third author (JES).

### Statistical analyses

To estimate age-specific dementia mortality rates, we modelled mortality rates for dementia in people with and without diabetes, using age and calendar time as continuous variables. Data were split by calendar time (into 1 year intervals) and age group (into 10 year intervals). Each interval was assigned the midpoint value of the calendar year and age group. Rate models with a Poisson likelihood were used to estimate dementia mortality, with log person-years as offset. Age–period–cohort models were fitted using cubic splines, for which knots were evenly spaced at quantiles of age, period (calendar time) and cohort (period minus age). Dementia mortality rates were estimated for attained ages 60, 70, 80 and 90 years for both sexes combined, and then separately for men and women. Ages below 60 years were excluded because mortality due to dementia was uncommon at these ages. The 95% CIs were calculated using the Wald method. We also calculated dementia mortality rate ratios, comparing individuals with and without diabetes, such that a mortality rate ratio of 2.0 indicates that dementia mortality rate among individuals with diabetes is twice that of those without diabetes. Age-specific dementia mortality rate ratios were estimated using a Poisson regression model that included sex, spline terms for calendar time, and an interaction between spline terms for age and diabetes status.

To estimate the 5 year change in dementia mortality rates by age, two Poisson regression models were fitted: (1) with an interaction between spline terms for age and a log-linear effect of calendar time; and (2) with a spline term for age and the product of log-linear effects of age and calendar time.

Further analyses included estimation of mortality rates and mortality rate ratios for dementia subtypes, namely Alzheimer’s disease, vascular dementia and unspecified dementia. Age-standardised mortality rates were calculated using direct standardisation to the total diabetes population, obtained by pooling person-years over all years of follow-up from the consortium. Rates were stratified by sex and presented by calendar time using age–period–cohort models, as described above. Standardised rates were restricted to ages 40–89 years due to the small number of deaths due to dementia subtypes in age groups outside this range.

We also fitted a set of age–period models with smooth functions for age and a linear effect of calendar time for each data source. These models were used to estimate the 5 year per cent change in the cause-specific mortality rates for dementia subtypes over each period, both overall and stratified by sex. For the mortality rate ratio for dementia subtypes, we calculated the mortality rate ratio for people with vs without diabetes by fitting a Poisson model with spline terms for age and calendar time, binary effects of sex and diabetes status, and interaction between diabetes status and calendar time. We used these models to predict the mortality rate ratio for each dementia subtype, jurisdiction and calendar year. We also fitted a set of models with similar parameterisation, except with a log-linear effect of calendar time, to provide an overall summary of the 5 year change in mortality rate ratio by dementia subtype for each jurisdiction.

Since the databases were very large, we applied an algorithm for classifying trends that did not rely only on the usual understanding of statistical significance, but also on the magnitude of the estimated change in dementia mortality rates/mortality rate ratios. As such, small but formally statistically significant changes could be labelled as stable. A null interval of −5.0% to +5.0% per 5 years was chosen for changes in rates/mortality rate ratios. If the CI for the change in dementia mortality rate/mortality rate ratio was entirely outside the null interval, the trend was labelled as increasing/decreasing. If the CI was entirely within the null interval, the trend was labelled as stable. All other cases were labelled uncertain. An illustration of these definitions is shown in ESM Fig. 1.

Data management, statistical analyses and graphics generation were conducted using Stata 17.0 (StataCorp, College Station, TX, USA). For all data sources, data were collected as part of routine clinical care and individual-level data were only visible to data custodians. This study was approved by the Human Research Ethics Committee of Alfred Health, Melbourne, VIC, Australia.

## Results

### Population characteristics

Descriptions of the populations with and without diabetes from the seven jurisdictions are summarised in Table 1. Data sources included administrative databases (3/7) and national registries (4/7). A total of 114,559 dementia deaths were reported for individuals with diabetes aged 60 years and above in over 42 million person-years of follow-up. For the non-diabetes group, 589,706 dementia deaths were reported for ages 60+ years in 244 million person-years of follow-up.

### Age-specific trends in dementia mortality among people with and without diabetes

**Fig. 1:**
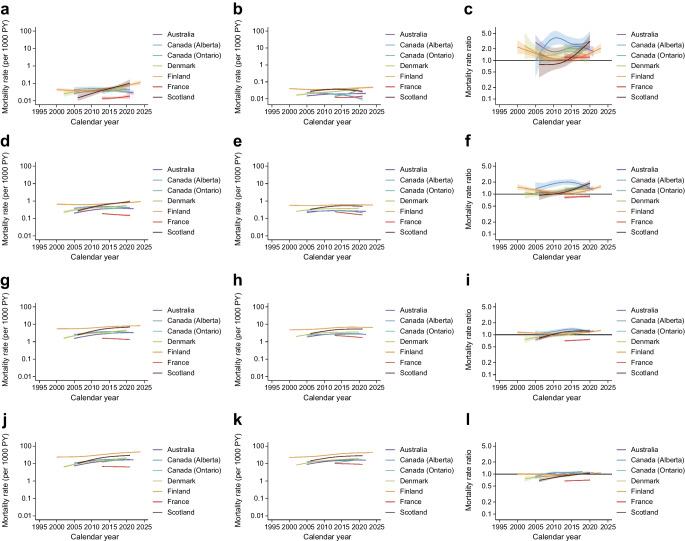
(**a**, **b**, **d**, **e**, **g**, **h**, **j**, **k**) Dementia mortality rates by calendar time for people with and without diabetes at 60 (**a**, **b**, respectively), 70 (**d**, **e**), 80 (**g**, **h**) and 90 years of age (**j**, **k**). (**c**, **f**, **i**, **l**) Dementia mortality rate ratio by calendar time for people with diabetes vs those without diabetes at 60 (**c**), 70 (**f**), 80 (**i**) and 90 (**l**) years of age. PY, person-years of follow-up. Data are expressed as mean (line) and 95% CI (shaded area). *y*-axes are on a log scale

Figure 1 shows the dementia mortality rates for people with and without diabetes and mortality rate ratios for people with vs without diabetes at 60, 70, 80 and 90 years of age in the seven jurisdictions. The mean per cent changes in dementia mortality rates over a 5 year period for the different ages and jurisdictions are shown in Table 2.

**Table 2:** Mean 5 year per cent change (95% CI) in dementia mortality rates by jurisdiction, age and diabetes status

Jurisdiction	Diabetes	No diabetes
Age 60 years		
Australia	−1.8 (−12.3, 10.0)^a^	−0.5 (−5.2, 4.5)^a^
Canada (Alberta)	−13.8 (−29.2, 5.0)^a^	−19.8 (−28.9, −9.6)^b^
Canada (Ontario)	36.2 (−12.8, 112.8)^a^	32.9 (2.3, 72.6)^a^
Denmark	8.0 (−11.4, 31.7)^a^	12.4 (5.1, 20.1)^c^
Finland	−4.5 (−11.5, 3.1)^a^	−6.7 (−9.9, −3.4)^a^
France	9.5 (−14.9, 40.9)^a^	9.5 (−0.0, 20.0)^a^
Scotland	44.3 (15.6, 80.1)^c^	−20.5 (−26.4, −14.0)^b^
Age 70 years		
Australia	15.6 (10.0, 21.4)^c^	6.0 (3.8, 8.2)^a^
Canada (Alberta)	−0.5 (−8.9, 8.7)^a^	−3.4 (−8.1, 1.6)^a^
Canada (Ontario)	22.3 (0.6, 48.7)^a^	11.0 (−0.5, 23.9)^a^
Denmark	23.1 (13.3, 33.8)^c^	13.1 (10.1, 16.3)^c^
Finland	4.1 (0.9, 7.4)^a^	2.4 (1.0, 3.9)^d^
France	−4.8 (−14.4, 5.9)^a^	−8.4 (−11.8, −4.9)^a^
Scotland	42.5 (30.9, 55.2)^c^	8.8 (5.4, 12.3)^c^
Age 80 years		
Australia	24.1 (21.3, 27.0)^c^	11.7 (10.7, 12.8)^c^
Canada (Alberta)	11.9 (7.2, 16.8)^c^	7.6 (5.0, 10.4)^c^
Canada (Ontario)	21.0 (11.0, 31.9)^c^	10.1 (4.6, 15.9)^c^
Denmark	29.1 (24.2, 34.2)^c^	17.1 (15.6, 18.7)^c^
Finland	13.9 (12.6, 15.3)^c^	11.1 (10.4, 11.8)^c^
France	−10.4 (−14.1, −6.4)^b^	−15.2 (−16.5, −13.8)^b^
Scotland	42.4 (37.4, 47.4)^c^	31.4 (28.9, 33.9)^c^
Age 90 years		
Australia	22.1 (19.8, 24.4)^c^	15.4 (14.7, 16.1)^c^
Canada (Alberta)	13.7 (10.1, 17.5)^c^	11.3 (9.5, 13.2)^c^
Canada (Ontario)	35.5 (27.1, 44.5)^c^	29.0 (24.8, 33.4)^c^
Denmark	32.0 (27.9, 36.1)^c^	22.9 (21.7, 24.1)^c^
Finland	18.3 (17.2, 19.4)^c^	16.5 (16.0, 17.0)^c^
France	−5.4 (−8.5, −2.1)^a^	−8.8 (−9.8, −7.9)^b^
Scotland	36.1 (32.0, 40.3)^c^	20.7 (19.0, 22.5)^c^

^a^Uncertain: 95% CIs for 5 year per cent change in mortality rates crossover 5 or −5

^b^Decreasing: 95% CIs for 5 year per cent change in mortality rates <−5

^c^Increasing: 95% CIs for 5 year per cent change in mortality rates >5

^d^Stable: 95% CIs for 5 year per cent change in mortality rates lie within −5 to 5

At younger ages (60 and 70 years), the trends in dementia mortality over time were mixed, such that some jurisdictions reported increases in dementia mortality, while others reported no clear trends. No declines in dementia mortality were seen for individuals with diabetes. In contrast, Alberta (Canada) and Scotland specifically observed declines in dementia mortality over time for those without diabetes at 60 years of age.

For individuals aged 80 and 90 years, most jurisdictions reported increases in dementia mortality over time for both individuals with and without diabetes. However, the magnitude of the increases in dementia mortality rates was generally greater for those with diabetes. For example, at 80 years of age, Australia reported a mean increase in dementia mortality rate of 24.1% (95% CI 21.3, 27.0) and 11.7% (10.7, 12.8) per 5 years for those with and without diabetes, respectively. France was the only jurisdiction to report declines in dementia mortality rates over time, with significant reductions of 10.4% and 15.2% per 5 years for individuals with and without diabetes, respectively, at 80 years of age. Further, most jurisdictions reported higher dementia mortality rates in individuals with diabetes compared with those without at 60 and 70 years of age. However, for those aged 80+ years, the mortality rate ratios were less than 1.0 for many jurisdictions.

Analyses using alternative thresholds of 3% and 7% to define meaningful differences in dementia prevalence yielded consistent findings (ESM Tables 2, 3).

### Age-specific trends in dementia mortality among people with and without diabetes stratified by sex

ESM Figs 2 and 3 present the sex-specific dementia mortality rates for those with and without diabetes and mortality rate ratios for those with vs without diabetes in the seven jurisdictions at 60, 70, 80 and 90 years of age. The mean per cent changes in dementia mortality rates over a 5 year period are shown in ESM Table 4.

No apparent increases or decreases in dementia mortality were observed for men aged 60 years. Conversely, at 80 and 90 years of age, increases in dementia mortality rates over time were reported by six jurisdictions. These increases ranged from 13.1% to 46.2% and 11.1% to 34.6% per 5 years for men with and without diabetes, respectively. Notably, the increases in dementia mortality rates were generally greater for men with diabetes than for those without. France was the only jurisdiction to report declines in dementia mortality for both groups at 80 and 90 years of age.

For women at younger ages (60 and 70 years), most jurisdictions reported no clear trends, with the exception of Alberta (Canada) and Scotland, which reported declines in dementia mortality for women without diabetes at 60 years of age. Consistent with the findings for men, most jurisdictions reported increases in dementia mortality at 80 and 90 years of age for women with and without diabetes, ranging from 10.4% to 40.3% and from 11.0% to 31.5%, respectively. However, the magnitude of the increases in dementia mortality rates was generally greater for women with diabetes than for women without diabetes.

Most jurisdictions reported mortality rate ratios greater than 1.0 at 60 and 70 years of age, indicating that the dementia mortality was higher in men and women with diabetes than in those without diabetes. At older ages, no meaningful excess in dementia mortality was apparent for men or women with diabetes compared with those without diabetes.

### Trends in mortality for dementia subtypes among individuals with and without diabetes

**Fig. 2:**
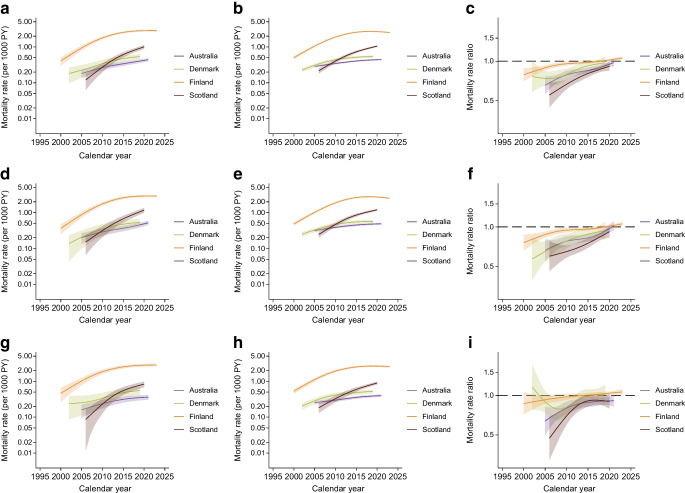
(**a**–**c**) Age-standardised Alzheimer’s disease mortality rates for people with (**a**) and without diabetes (**b**) and mortality rate ratios for people with diabetes vs those without diabetes (**c**) by calendar year in people aged 40–89 years. (**d**–**f**) Mortality rates for women with (**d**) and without diabetes (**e**) and mortality rate ratios (**f**). (**g**–**i**) Mortality rates for men with (**g**) and without diabetes (**h**) and mortality rate ratios (**i**). Data are expressed as mean (line) and 95% CI (shaded area). *y*-axes are on a log scale

Figure 2 shows the age-standardised mortality rates and mortality rate ratios for Alzheimer’s disease in Australia, Denmark, Finland and Scotland. Mortality due to Alzheimer’s disease increased across all four jurisdictions in individuals with and without diabetes. However, Alzheimer’s disease mortality was lower in individuals with diabetes. Despite this, increasing trends in the mortality rate ratios over time were observed. Notably, the mortality due to Alzheimer’s disease increased more rapidly in women with diabetes, compared with those without diabetes, as well as in both men and women combined in Australia and Scotland. This was evident in the increasing 5 year per cent change in the mortality rate ratios (ESM Table 5). In contrast, the uncertain or stable trends in the mortality rate ratios over time reported by Denmark and Finland suggested similar increases in Alzheimer’s disease mortality among individuals with and without diabetes.

**Fig. 3:**
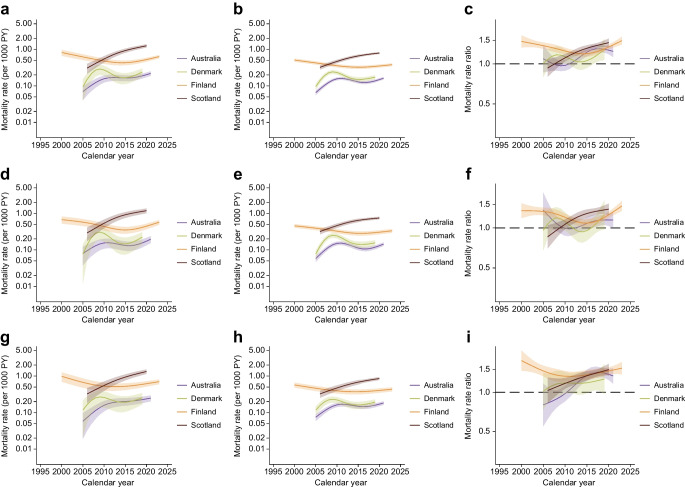
(**a**–**c**) Age-standardised vascular dementia mortality rates for people with (**a**) and without diabetes (**b**) and mortality rate ratios for people with diabetes vs those without diabetes (**c**) by calendar year in people aged 40–89 years. (**d**–**f**) Mortality rates for women with (**d**) and without diabetes (**e**) and mortality rate ratios (**f**). (**g**–**i**) Mortality rates for men with (**g**) and without diabetes (**h**) and mortality rate ratios (**i**). Data are expressed as mean (line) and 95% CI (shaded area). *y*-axes are on a log scale

Vascular dementia mortality increased over time for both the diabetes and non-diabetes groups, except in Finland, where the trends remained stable or uncertain (Fig. 3 and ESM Table 6). The mortality rate ratios were generally greater than 1.0, indicating a higher vascular dementia mortality among those with diabetes relative to those without. Further, the increasing trends in the mortality rate ratios suggested that the increases in vascular dementia mortality were greater among men with diabetes in Australia, and both men and women with diabetes in Scotland, compared with people without a diagnosis of diabetes in these populations.

For unspecified dementia, increasing mortality rates over time were observed in Australia and Denmark for both the diabetes and non-diabetes groups (ESM Fig. 4, ESM Table 7). In Finland, unspecified dementia mortality trends showed a decline for both individuals with and without diabetes, while in Scotland, trends were uncertain for individuals with diabetes and declining/stable for those without diabetes. For Denmark and Scotland, the unspecified dementia mortality rate increased more rapidly in individuals with diabetes than in those without diabetes.

### Risk of bias assessment of data sources

The risk of bias assessment revealed that two out of the seven jurisdictions received the highest possible score of 9 (ESM Table 8). The lowest score assigned was 6, which was given to one jurisdiction. The median quality score across all jurisdictions was 8, with an IQR of 7–9. These findings suggest generally high-quality data sources, with minor variability in data quality across jurisdictions.

## Discussion

The present study, one of the largest global analyses conducted to date, reports age- and sex-specific secular trends in dementia mortality in people with and without diabetes between 2000 and 2023. We analysed 704,265 deaths recorded with dementia as the underlying cause and approximately 286 million person-years of follow-up among people aged 60+ years. Our findings revealed significant age- and jurisdiction-specific variations in dementia mortality trends, with the trends generally being similar between men and women.

At 60 and 70 years of age, individuals with and without diabetes showed mixed trends, with increases in dementia mortality in some regions and uncertain trends in others. Of note, no jurisdiction reported declines in dementia mortality at age 60 and 70 years for those with diabetes. In contrast, Alberta (Canada) and Scotland specifically observed declining dementia mortality in those without diabetes at 60 years of age.

In individuals at 80 and 90 years of age, most jurisdictions showed increases in dementia mortality rates, with larger increases seen in people with diabetes than in those without diabetes. An exception was noted for France, where the trends were mainly declining at 80 and 90 years of age. The declining dementia mortality rates observed in France, in contrast to increasing trends in most other jurisdictions, might reflect differences in death certification and coding practices. In older adults with chronic conditions, the underlying cause of death attribution could vary between countries, with some certification systems more likely to assign deaths to acute conditions such as infections rather than dementia.

Scotland was the only jurisdiction with consistent increases in dementia mortality across all ages for individuals with diabetes, while Denmark was the only jurisdiction with increases in dementia mortality for those without diabetes at all ages.

It is noteworthy that most jurisdictions found the mortality rate ratios to be greater than 1.0 for individuals at 60 and 70 years of age, indicating higher relative dementia mortality in individuals with diabetes. Conversely, for individuals aged 80 years and older, most jurisdictions reported mortality rate ratios <1.0, suggesting that individuals with diabetes were less likely to die from dementia than those without. We believe that the similar dementia mortality between people with and without diabetes in older age groups may be attributable to healthy survivor bias or differences in death certification and coding practices. Alongside declining relative dementia mortality by diabetes status with increasing age, absolute dementia mortality generally appeared to increase more rapidly over time in people with diabetes relative to those without.

A novel feature of our work is the examination of mortality trends for specific dementia subtypes. Except for Finland, which reported stable or uncertain trends in vascular dementia mortality and decreasing trends in unspecified dementia mortality, all other jurisdictions observed increasing mortality rates for dementia subtypes for both diabetes and non-diabetes groups. These trends appeared more consistent than for total dementia mortality, likely because cause-specific mortality was examined across the broader age group of 40–89 years, while total dementia mortality was limited to analysis at 60, 70, 80 and 90 years of age. Additionally, improvements in diagnostic specificity over time may have led to an increase in coding of specific dementia types (e.g. Alzheimer’s disease or vascular dementia), contributing to apparent rises in cause-specific mortality alongside potential declines in unspecified dementia mortality.

Interestingly, for mortality from Alzheimer’s disease and unspecified dementia, rates were lower in people with diabetes than in those without diabetes, as indicated by the mortality rate ratios. In contrast, vascular dementia mortality was higher among individuals with diabetes than among those without diabetes. This elevated vascular dementia mortality may reflect a greater cerebrovascular disease burden in people with diabetes. Type 2 diabetes can contribute to the development of vascular dementia through mechanisms such as hypoxia, ischaemia and blood–brain barrier leakage, which promote microvascular damage and cerebrovascular dysfunction [14, 15]. Previous studies have similarly reported a greater burden of vascular dementia compared with Alzheimer’s disease in individuals with diabetes [4, 7, 16, 17].

Recent work from our group indicates that age- and sex-standardised dementia mortality has increased in both people with and without diabetes aged 40–89 years across all jurisdictions, except for France [18]. The increase in overall dementia mortality was greater among individuals with diabetes in Denmark and Scotland. In contrast, in France, dementia mortality declined among individuals without diabetes, while there were no obvious time trends among those with diabetes.

Overall, our findings align with a broader body of evidence suggesting that dementia mortality is rising globally, particularly in high-income countries where dementia is now the second-leading cause of mortality [19–21]. The rise in dementia mortality is often attributed to population ageing and declines in CVD-related mortality, underscoring the substantial global burden [19]. These trends should also be interpreted alongside previously reported declines in all-cause and cardiovascular mortality among people with diabetes in these jurisdictions [2]. Further, the observed rise in dementia mortality may reflect the successful treatment of other causes of mortality (e.g. CVD and cancers), such that people are living longer and later dying of neurodegenerative conditions. Thus, rising dementia mortality, particularly at older ages, may also reflect a reduction in competing risks of death. While we could not directly account for changes in other causes of death, our findings are consistent with our related multi-country analyses of proportional mortality across multiple causes, which showed declining proportional mortality from CVD alongside increasing proportional mortality from dementia in most jurisdictions [18].

Our findings also revealed that the rise in dementia mortality rates is more pronounced in older individuals and those with diabetes, although this may partly reflect the larger number of events in these groups. The relatively larger increases in dementia mortality rates in individuals with diabetes at any given age may suggest that the rise is not driven by population ageing, since our age-specific analysis showed that individuals of the same age (e.g. 80 years) were more likely to die from dementia at the end of the observation period than at the beginning. This corroborates the current evidence of a changing landscape of diabetes complications and diversification of causes of death among those with diabetes [22, 23]. Given that there is now a greater survival of people with diabetes, there exists a greater opportunity for age-related complications to develop. It is interesting to note that in recent decades, absolute mortality has declined in people with diabetes worldwide [22, 23]. Further, there has been a decline in the proportion of mortality from traditional causes among people with diabetes, with concomitant large increases in the proportion of mortality from emerging causes, including dementia, highlighting the need for comprehensive monitoring of a diverse range of diabetes-associated complications [22, 23]. Thus, focused dementia prevention and care strategies for people with diabetes might be beneficial considering the rising trends in diabetes-related dementia mortality. While the increasing dementia mortality in the setting of diabetes is concerning, the underlying mechanisms remain poorly understood and adequate treatments are still unavailable. This highlights the need for future research to better elucidate the biological factors driving this trend and to develop lifestyle and/or pharmaceutical interventions to prevent dementia, especially in people with diabetes.

### Strengths and limitations

This is the first multi-country, population-based analysis to report age- and sex-specific secular trends in dementia mortality and its subtypes in people with and without diabetes using a strict protocol. However, several limitations should be considered. The analysis only included high-income countries, as data from low- and middle-income countries were not available and patterns in these countries may be different. Sex was derived from administrative records and information on gender identity was unavailable; therefore, the findings only reflect differences between male and female sex and may not be generalisable to gender-diverse populations. The diabetes population comprised diagnosed diabetes cases only and the definition of these varied across jurisdictions.

Death certification practices could be an important source of uncertainty. Varying coding practices in underlying causes of death between jurisdictions and over time may affect recorded rates of dementia mortality, as some deaths among people with dementia may be attributed to acute conditions such as pneumonia rather than dementia itself [4]. Dementia is also frequently recorded as a contributing rather than underlying cause of death, which may lead to underestimation of dementia mortality rates. Conversely, changes in certification practices may have improved the recognition and reporting of dementia over time [20]. Dementia subtype classification based on death certificates should be interpreted cautiously, as accurate differentiation generally requires neuropathological confirmation [25, 26]. Together, the factors relating to certification and coding practices may contribute to cross-country variations and observed temporal trends.

Additionally, the observed increases in dementia mortality in recent years could also be influenced by reduced reporting of comorbid conditions on death certificates, particularly CVD, in high-income countries [20]. We were also unable to determine whether rising dementia mortality rates reflect increasing prevalence of dementia or changes in mortality risk among people already living with dementia, as information on dementia diagnosis and duration before death was not available. Further, increased specification of dementia subtypes among people with multiple long-term conditions could also have influenced mortality rates over time. For example, reductions in mortality for unspecified dementia in Finland probably reflect a trend towards coding as Alzheimer’s disease or vascular dementia. In addition, dementia deaths at younger ages, particularly around 60 years, were relatively uncommon in some jurisdictions, resulting in greater variability.

### Conclusions

In summary, this large-scale, multi-national study indicates that the dementia mortality rate is rising for individuals with and without diabetes, with rises being greater at 80 and 90 years compared with 60 and 70 years of age and among those with (vs without) diabetes in high-income jurisdictions. Specific jurisdictions, such as Scotland, consistently reported increasing trends in dementia mortality at all ages for individuals with diabetes only. These trends reflect a shift in the causes of mortality, particularly among individuals with diabetes, with possible reductions in cardiovascular mortality and a growing contribution of dementia. Overall, the findings reinforce the need for developing strategies and/or therapeutics to delay or prevent the onset of dementia as well as the use of cost-effective interventions for high-risk ageing populations such as those with diabetes.

## Supplementary Information

Below is the link to the electronic supplementary material.

ESM (PDF 548 KB)

## Data Availability

Aggregated data can be made available upon reasonable request to the senior authors subject to approval from the data custodians.
