# Supplementary material for: Age- and sex-specific trends in dementia mortality among people with and without diabetes: a multi-country population-based analysis
Source: Diabetologia. 2026 Apr 21;69(7):1898–910. doi: 10.1007/s00125-026-06725-2 (PMC13236754; doi:10.1007/s00125-026-06725-2)
Supplement: Supplementary file 1 — ESM (PDF 548 KB) [file 125_2026_6725_MOESM1_ESM.pdf]

Electronic supplementary material (ESM)

ESM Methods

Quality of included data

We used a modified Newcastle-Ottawa Quality Assessment Scale.

The scale includes items that assess the representativeness of the data sources, sample size at each time point, the method of defining diabetes, whether people with gestational diabetes were excluded, completeness of the number of data points reported and the assessment of the outcome (death). The maximum score was 9.0.

Selection

1. Representativeness of the general population (sampling frame).

    a) National scheme with ≥80% coverage of national population (2 points)
    b) Random sample from national health insurance or national population-based survey with ≥80% response rate (1 point)
    c) Regional representative or national scheme with <80% coverage of national population (0 points)

2. Assessment of diabetes status.

    a) By blood glucose measurement (FPG, OGTT, HbA1c) or by multiple approaches / administrative algorithm where 2 or more criteria are used (2 points)
    b) Clinical diagnosis (e.g. ICD code or physician-diagnosed) (1 point)
    c) Glucose-lowering medications or self-report of physician-diagnosed diabetes (0 points)

3. Exclusion of gestational diabetes.

    a) Yes (1 point)
    b) No (0 points)

4. Sample size at each time point.

a) >10,000 (1 point)

b) ≤10,000 (0 points)

Outcome

1. Assessment of outcome.

a) By record linkage (1 point) e.g. national death registry

b) Other – regular follow-up (0 points)

Completeness of trend data

1. How many time points are provided?

a) ≥10 (2 points)

b) 6 – 9 (1 point)

c) <6 (0 points)

ESM Table 1. Diabetes definitions by data source.

**Jurisdiction**	**Diabetes definition**	**Gestational diabetes excluded**
Australia	Clinical diagnosis certified by a doctor, nurse or credentialed diabetes educator	Yes
Canada (Alberta)	Algorithm incorporating ≥1 hospitalisations or ≥2 physician claims with evidence of diabetes within 2 years	Yes
Canada (Ontario)	Algorithm incorporating ≥1 hospitalisations or ≥2 physician claims with evidence of diabetes within 2 years	Yes
Denmark	Algorithm incorporating clinical diagnosis (ICD-10 codes) from the hospitalisations, prescription of glucose-lowering medications, clinical and billing records	Yes
Finland	Algorithm incorporating clinical diagnosis (ICD-10 codes) from the Care Register for Health Care, Register of Primary Health Care Visits, Medical Birth Register and Causes of Death Statistics and prescription of glucose-lowering medications	Yes
France	People who have been reimbursed for at least 3 glucose-lowering medications in the year, or 2 in cases of large packaging	No
Scotland	Clinical diagnosis using the Read coding system (ICD-10 codes)	Yes

ICD = International Classification of Diseases; ICD-10 = International Classification of Diseases, 10th edition.

ESM Table 2. Mean 5 year percent changes in dementia mortality rates by jurisdiction, age and diabetes status using a 3% threshold for meaningful change.

**Jurisdiction**	**5 year mean percent change (95% CI)**	
	Diabetes	Non-diabetes
**Age 60**		
Australia	-1.8 (-12.3, 10.0)	-0.5 (-5.2, 4.5)
Canada (Alberta)	-13.8 (-29.2, 5.0)	-19.8 (-28.9, -9.6)
Canada (Ontario)	36.2 (-12.8, 112.8)	32.9 (2.3, 72.6)
Denmark	8.0 (-11.4, 31.7)	12.4 (5.1, 20.1)
Finland	-4.5 (-11.5, 3.1)	-6.7 (-9.9, -3.4)
France	9.5 (-14.9, 40.9)	9.5 (-0.0, 20.0)
Scotland	44.3 (15.6, 80.1)	-20.5 (-26.4, -14.0)
**Age 70**		
Australia	15.6 (10.0, 21.4)	6.0 (3.8, 8.2)
Canada (Alberta)	-0.5 (-8.9, 8.7)	-3.4 (-8.1, 1.6)
Canada (Ontario)	22.3 (0.6, 48.7)	11.0 (-0.5, 23.9)
Denmark	23.1 (13.3, 33.8)	13.1 (10.1, 16.3)
Finland	4.1 (0.9, 7.4)	2.4 (1.0, 3.9)
France	-4.8 (-14.4, 5.9)	-8.4 (-11.8, -4.9)
Scotland	42.5 (30.9, 55.2)	8.8 (5.4, 12.3)
**Age 80**		
Australia	24.1 (21.3, 27.0)	11.7 (10.7, 12.8)
Canada (Alberta)	11.9 (7.2, 16.8)	7.6 (5.0, 10.4)
Canada (Ontario)	21.0 (11.0, 31.9)	10.1 (4.6, 15.9)
Denmark	29.1 (24.2, 34.2)	17.1 (15.6, 18.7)
Finland	13.9 (12.6, 15.3)	11.1 (10.4, 11.8)
France	-10.4 (-14.1, -6.4)	-15.2 (-16.5, -13.8)
Scotland	42.4 (37.4, 47.4)	31.4 (28.9, 33.9)
**Age 90**		
Australia	22.1 (19.8, 24.4)	15.4 (14.7, 16.1)
Canada (Alberta)	13.7 (10.1, 17.5)	11.3 (9.5, 13.2)
Canada (Ontario)	35.5 (27.1, 44.5)	29.0 (24.8, 33.4)
Denmark	32.0 (27.9, 36.1)	22.9 (21.7, 24.1)
Finland	18.3 (17.2, 19.4)	16.5 (16.0, 17.0)
France	-5.4 (-8.5, -2.1)	-8.8 (-9.8, -7.9)
Scotland	36.1 (32.0, 40.3)	20.7 (19.0, 22.5)

Stable	95% CIs for 5 year percent change in mortality rates lie within -3 to 3
Uncertain	95% CIs for 5 year percent change in mortality rates cross over 3 or -3
Increasing	95% CIs for 5 year percent change in mortality rates > 3
Decreasing	95% CIs for 5 year percent change in mortality rates < -3

ESM Table 3. Mean 5 year percent changes in dementia mortality rates by jurisdiction, age and diabetes status using a 7% threshold for meaningful change.

Jurisdiction	5 year mean percent change (95% CI)	
	Diabetes	Non-diabetes
**Age 60**		
Australia	-1.8 (-12.3, 10.0)	-0.5 (-5.2, 4.5)
Canada (Alberta)	-13.8 (-29.2, 5.0)	-19.8 (-28.9, -9.6)
Canada (Ontario)	36.2 (-12.8, 112.8)	32.9 (2.3, 72.6)
Denmark	8.0 (-11.4, 31.7)	12.4 (5.1, 20.1)
Finland	-4.5 (-11.5, 3.1)	-6.7 (-9.9, -3.4)
France	9.5 (-14.9, 40.9)	9.5 (-0.0, 20.0)
Scotland	44.3 (15.6, 80.1)	-20.5 (-26.4, -14.0)
**Age 70**		
Australia	15.6 (10.0, 21.4)	6.0 (3.8, 8.2)
Canada (Alberta)	-0.5 (-8.9, 8.7)	-3.4 (-8.1, 1.6)
Canada (Ontario)	22.3 (0.6, 48.7)	11.0 (-0.5, 23.9)
Denmark	23.1 (13.3, 33.8)	13.1 (10.1, 16.3)
Finland	4.1 (0.9, 7.4)	2.4 (1.0, 3.9)
France	-4.8 (-14.4, 5.9)	-8.4 (-11.8, -4.9)
Scotland	42.5 (30.9, 55.2)	8.8 (5.4, 12.3)
**Age 80**		
Australia	24.1 (21.3, 27.0)	11.7 (10.7, 12.8)
Canada (Alberta)	11.9 (7.2, 16.8)	7.6 (5.0, 10.4)
Canada (Ontario)	21.0 (11.0, 31.9)	10.1 (4.6, 15.9)
Denmark	29.1 (24.2, 34.2)	17.1 (15.6, 18.7)
Finland	13.9 (12.6, 15.3)	11.1 (10.4, 11.8)
France	-10.4 (-14.1, -6.4)	-15.2 (-16.5, -13.8)
Scotland	42.4 (37.4, 47.4)	31.4 (28.9, 33.9)
**Age 90**		
Australia	22.1 (19.8, 24.4)	15.4 (14.7, 16.1)
Canada (Alberta)	13.7 (10.1, 17.5)	11.3 (9.5, 13.2)
Canada (Ontario)	35.5 (27.1, 44.5)	29.0 (24.8, 33.4)
Denmark	32.0 (27.9, 36.1)	22.9 (21.7, 24.1)
Finland	18.3 (17.2, 19.4)	16.5 (16.0, 17.0)
France	-5.4 (-8.5, -2.1)	-8.8 (-9.8, -7.9)
Scotland	36.1 (32.0, 40.3)	20.7 (19.0, 22.5)

Stable	95% CIs for 5 year percent change in mortality rates lie within -7 to 7
Uncertain	95% CIs for 5 year percent change in mortality rates cross over 7 or -7
Increasing	95% CIs for 5 year percent change in mortality rates > 7
Decreasing	95% CIs for 5 year percent change in mortality rates < -7

ESM Table 4. Mean 5 year percent changes in dementia mortality rates by jurisdiction, age, diabetes status and sex.

Jurisdiction	5 year mean percent change for men (95% CI)		5 year mean percent change for women (95% CI)	
	Diabetes	Non-diabetes	Diabetes	Non-diabetes
**Age 60**				
Australia	-5.2 (-18.5, 10.2)	-2.7 (-9.4, 4.5)	4.1 (-12.6, 24.1)	1.4 (-5.4, 8.6)
Alberta (Canada)	-17.4 (-37.3, 8.8)	-16.6 (-30.6, 0.4)	-5.7 (-29.7, 26.5)	-24.6 (-35.9, -11.2)
Ontario (Canada)	-12.8 (-52.3, 59.4)	15.3 (-22.0, 70.3)	137.0 (15.2, 387.3)	49.0 (4.2, 113.1)
Denmark	16.3 (-11.9, 53.7)	2.5 (-7.0, 13.0)	3.0 (-23.3, 38.1)	22.0 (11.3, 33.8)
Finland	-7.5 (-16.5, 2.4)	-5.8 (-10.4, -0.9)	-0.6 (-11.8, 12.0)	-7.6 (-11.9, -3.0)
France	-10.4 (-35.5, 24.3)	12.6 (-1.2, 28.3)	49.1 (-0.7, 123.9)	7.8 (-5.2, 22.6)
Scotland	37.5 (2.4, 84.7)	-15.2 (-24.6, -4.7)	51.0 (7.5, 112.0)	-25.1 (-32.6, -16.7)
**Age 70**				
Australia	13.3 (6.2, 20.9)	5.4 (2.3, 8.6)	19.2 (10.3, 28.7)	6.3 (3.3, 9.4)
Alberta (Canada)	-5.6 (-16.2, 6.4)	0.8 (-6.5, 8.7)	4.9 (-8.1, 19.7)	-6.3 (-12.5, 0.4)
Ontario (Canada)	15.0 (-11.2, 49.1)	8.3 (-7.8, 27.2)	33.1 (-1.5, 79.8)	13.8 (-2.1, 32.1)
Denmark	20.2 (7.0, 35.0)	8.8 (4.6, 13.2)	25.1 (10.4, 41.7)	17.2 (12.8, 21.7)
Finland	2.0 (-2.1, 6.4)	2.2 (0.2, 4.4)	5.8 (0.9, 11.0)	2.1 (0.1, 4.2)
France	-12.9 (-24.1, -0.1)	-9.3 (-14.0, -4.3)	8.3 (-8.4, 28.0)	-7.7 (-12.5, -2.6)
Scotland	38.7 (23.8, 55.3)	8.6 (3.5, 13.9)	47.3 (29.6, 67.4)	8.2 (3.7, 13.0)
**Age 80**				
Australia	21.5 (17.5, 25.7)	12.8 (11.1, 14.4)	26.8 (22.8, 30.9)	11.2 (9.9, 12.5)
Alberta (Canada)	13.4 (6.6, 20.6)	7.8 (3.5, 12.1)	10.4 (4.0, 17.2)	7.3 (3.9, 10.9)
Ontario (Canada)	26.1 (11.2, 43.0)	14.8 (5.8, 24.6)	17.9 (4.6, 32.8)	7.4 (0.6, 14.7)
Denmark	25.5 (18.3, 33.1)	16.8 (14.3, 19.4)	32.7 (26.0, 39.8)	17.7 (15.8, 19.7)
Finland	13.1 (11.0, 15.4)	11.1 (9.9, 12.3)	14.2 (12.5, 16.0)	11.0 (10.1, 11.8)
France	-12.9 (-18.3, -7.2)	-17.7 (-19.8, -15.5)	-7.3 (-12.6, -1.7)	-13.5 (-15.2, -11.8)
Scotland	46.2 (38.5, 54.3)	33.2 (29.2, 37.4)	40.3 (33.9, 47.0)	31.5 (28.4, 34.6)
**Age 90**				
Australia	22.3 (18.6, 26.2)	16.8 (15.4, 18.2)	22.8 (19.9, 25.8)	15.5 (14.6, 16.4)
Alberta (Canada)	20.2 (13.8, 26.9)	12.1 (8.7, 15.5)	10.4 (6.0, 15.1)	11.5 (9.3, 13.8)
Ontario (Canada)	29.1 (16.1, 43.6)	34.6 (26.7, 43.0)	38.0 (27.1, 49.8)	26.5 (21.5, 31.8)
Denmark	31.0 (23.8, 38.6)	23.6 (21.3, 25.9)	33.4 (28.4, 38.6)	22.9 (21.5, 24.3)
Finland	18.8 (16.6, 21.0)	17.8 (16.7, 18.8)	18.4 (17.1, 19.7)	16.3 (15.7, 17.0)
France	-9.1 (-14.3, -3.7)	-11.0 (-12.8, -9.2)	-3.3 (-7.4, 0.9)	-7.6 (-8.7, -6.4)
Scotland	39.1 (31.8, 46.8)	31.6 (28.1, 35.1)	35.5 (30.6, 40.6)	17.3 (15.4, 19.3)

Stable	95% CIs for 5 year percent change in mortality rates lie within -5 to 5
Uncertain	95% CIs for 5 year percent change in mortality rates cross over 5 or -5
Increasing	95% CIs for 5 year percent change in mortality rates > 5
Decreasing	95% CIs for 5 year percent change in mortality rates < -5

ESM Table 5. Mean 5 year percent change in Alzheimer's disease mortality rates and mortality rate ratios by jurisdiction, diabetes status and sex.

Jurisdiction	Sex	Mean 5 year percent change (95% CI) in mortality rates and mortality rate ratios		
		Change in mortality rates in people with diabetes	Change in mortality rates in people without diabetes	Change in MRRs for people with vs. without diabetes
Australia	Females	29.29 (24.10, 34.70)	15.91 (14.45, 17.39)	11.58 (6.91, 16.45)
	Males	25.68 (19.57, 32.12)	16.84 (14.68, 19.04)	7.97 (2.39, 13.86)
	Overall	27.83 (23.85, 31.95)	16.21 (14.99, 17.43)	10.22 (6.61, 13.94)
Denmark	Females	45.96 (36.12, 56.50)	30.55 (27.96, 33.18)	11.86 (4.03, 20.27)
	Males	33.7 (23.35, 44.93)	28.33 (24.76, 32.01)	4.2 (-4.33, 13.50)
	Overall	40.67 (33.44, 48.30)	29.81 (27.71, 31.94)	8.79 (2.94, 14.96)
Finland	Females	41.9 (40.05, 43.77)	40.12 (39.23, 41.02)	0.99 (-0.47, 2.46)
	Males	30.74 (28.39, 33.14)	31.33 (30.11, 32.56)	-0.46 (-2.47, 1.58)
	Overall	38.09 (36.63, 39.56)	37.31 (36.58, 38.03)	0.42 (-0.76, 1.61)
Scotland	Females	98.46 (85.22, 112.65)	70.55 (66.88, 74.31)	15.42 (7.37, 24.07)
	Males	94.1 (77.66, 112.07)	71.87 (66.13, 77.80)	12.63 (2.46, 23.80)
	Overall	96.81 (86.38, 107.82)	70.94 (67.83, 74.10)	14.62 (8.24, 21.38)

Stable	95% CIs for 5 year percent change in mortality rates/MRRs lie within -5 to 5
Uncertain	95% CIs for 5 year percent change in mortality rates/MRRs cross over 5 or -5
Increasing	95% CIs for 5 year percent change in mortality rates/MRRs > 5
Decreasing	95% CIs for 5 year percent change in mortality rates/MRRs < -5

ESM Table 6. Mean 5 year percent change in vascular dementia mortality rates and mortality rate ratios by jurisdiction, diabetes status and sex.

Jurisdiction	Sex	Mean 5 year percent change (95% CI) in mortality rates and mortality rate ratios		
		Change in mortality rates in people with diabetes	Change in mortality rates in people without diabetes	Change in MRRs for people with vs. without diabetes
Australia	Females	12.64 (5.74, 19.99)	10.4 (7.94, 12.91)	0.7 (-5.82, 7.67)
	Males	32.59 (24.48, 41.24)	14.6 (11.45, 17.83)	14.68 (7.06, 22.85)
	Overall	22.44 (17.10, 28.03)	12.05 (10.10, 14.03)	8.31 (3.26, 13.60)
Denmark	Females	18.15 (7.32, 30.08)	23.68 (19.70, 27.80)	-4.31 (-13.55, 5.92)
	Males	20.01 (7.47, 34.01)	19.19 (14.12, 24.49)	0.61 (-10.63, 13.27)
	Overall	18.95 (10.64, 27.90)	22.05 (18.89, 25.28)	-2.18 (-9.42, 5.64)
Finland	Females	-2.62 (-4.90, -0.29)	-5.23 (-6.46, -3.98)	1.73 (-0.97, 4.50)
	Males	-1.36 (-4.34, 1.71)	-4.39 (-6.14, -2.60)	2.05 (-1.52, 5.76)
	Overall	-2.15 (-3.97, -0.30)	-4.95 (-5.96, -3.92)	2.24 (0.07, 4.45)
Scotland	Females	56.53 (47.86, 65.71)	35.03 (31.93, 38.20)	13.65 (6.90, 20.84)
	Males	59.28 (49.59, 69.60)	40.67 (36.48, 45.00)	11.78 (4.27, 19.83)
	Overall	57.77 (51.25, 64.57)	37.1 (34.60, 39.65)	13.15 (8.08, 18.46)

Stable	95% CIs for 5 year percent change in mortality rates/MRRs lie within -5 to 5
Uncertain	95% CIs for 5 year percent change in mortality rates/MRRs cross over 5 or -5
Increasing	95% CIs for 5 year percent change in mortality rates/MRRs > 5
Decreasing	95% CIs for 5 year percent change in mortality rates/MRRs < -5

ESM Table 7. Mean 5 year percent change in unspecified dementia mortality rates and mortality rate ratios by jurisdiction, diabetes status and sex.

Jurisdiction	Sex	Mean 5 year percent change in mortality rates and mortality rate ratios (95% CI)		
		Change in mortality rates in people with diabetes	Change in mortality rates in people without diabetes	Change in MRRs for people with vs. without diabetes
Australia	Females	22.67 (19.38, 26.06)	14.83 (13.78, 15.90)	5.87 (2.88, 8.94)
	Males	17.45 (13.86, 21.16)	13.13 (11.63, 14.65)	3.6 (0.17, 7.15)
	Overall	20.4 (17.96, 22.89)	14.29 (13.42, 15.15)	4.86 (2.60, 7.16)
Denmark	Females	31.05 (25.48, 36.87)	18.11 (16.54, 19.71)	10.97 (6.04, 16.13)
	Males	28.03 (20.96, 35.51)	15.45 (13.13, 17.82)	10.85 (4.36, 17.74)
	Overall	29.93 (25.52, 34.50)	17.3 (15.99, 18.62)	10.97 (7.02, 15.06)
Finland	Females	-26.78 (-28.33, -25.19)	-29.2 (-29.97, -28.42)	2.82 (0.38, 5.31)
	Males	-23.44 (-26.14, -20.65)	-25.28 (-26.64, -23.90)	1.53 (-2.46, 5.68)
	Overall	-25.91 (-27.26, -24.54)	-28.2 (-28.87, -27.52)	2.52 (0.44, 4.64)
Scotland	Females	1.97 (-2.76, 6.94)	-9.02 (-10.61, -7.40)	10.94 (5.48, 16.67)
	Males	3.97 (-2.38, 10.73)	-1.91 (-4.63, 0.88)	5.17 (-1.81, 12.64)
	Overall	2.69 (-1.13, 6.67)	-7.07 (-8.45, -5.68)	9.7 (5.34, 14.24)

Stable	95% CIs for 5 year percent change in mortality rates/MRRs lie within -5 to 5
Uncertain	95% CIs for 5 year percent change in mortality rates/MRRs cross over 5 or -5
Increasing	95% CIs for 5 year percent change in mortality rates/MRRs > 5
Decreasing	95% CIs for 5 year percent change in mortality rates/MRRs < -5

ESM Table 8. Quality assessment of the included data sources.

Jurisdiction	Data source	Representativeness of population	Assessment of diabetes	Exclusion of gestational diabetes	Sample size at time points	Assessment of outcome	Completeness (no. of data points)	Total Score
Range of allocated points		0–2	0–2	0–1	0–1	0–1	0–2	9
Australia	National Diabetes Services Scheme linked to National Death Index, General Records of Incidence of Mortality	2	1	1	1	1	2	8
Canada (Alberta)	Population-level healthcare administrative database in Alberta	0	2	1	1	1	2	7
Canada (Ontario)	Population-level healthcare administrative database in Ontario	0	2	1	1	1	1	6
Denmark	National Patient Register, National Prescription Registry and National Health Service Register	2	2	1	1	1	2	9
Finland	FinDM (Diabetes in Finland) research database	2	2	1	1	1	2	9
France	Système National de Données de Santé (SNDS): French national health data system	2	2	0*	1	1	1	7
Scotland	Scottish Diabetes Research Network - National Diabetes Dataset 2021	2	1	1	1	1	2	8

*Although gestational diabetes was not excluded, this might not have impacted the analysis, which was restricted to 60+ population.

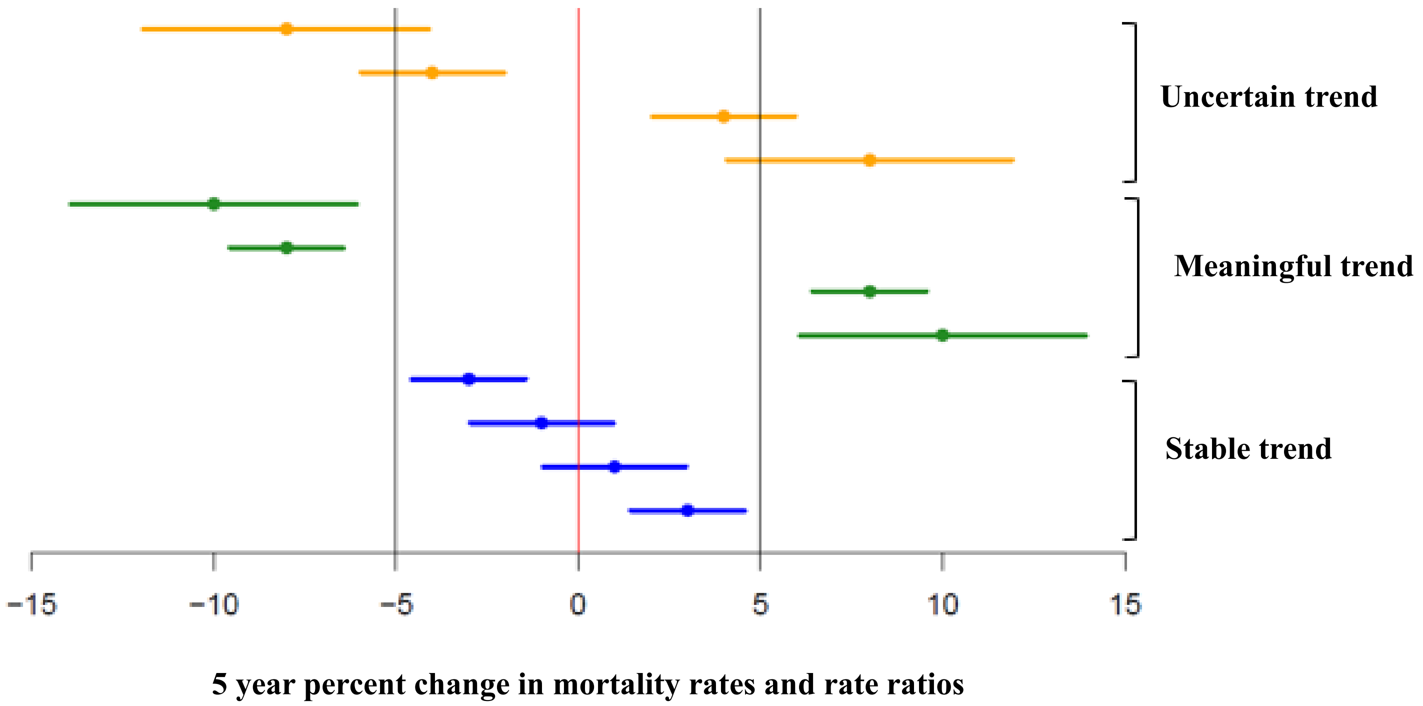

ESM Fig. 1. An illustration of percentage changes in mortality rates and rate ratios over 5 years that have been labelled as uncertain, meaningful or stable trends, using a null interval of –5 per five years to 5 per five years. Estimate refers to the trend in a rate over time, together with its confidence interval (spokes originating from the effect estimate dots).

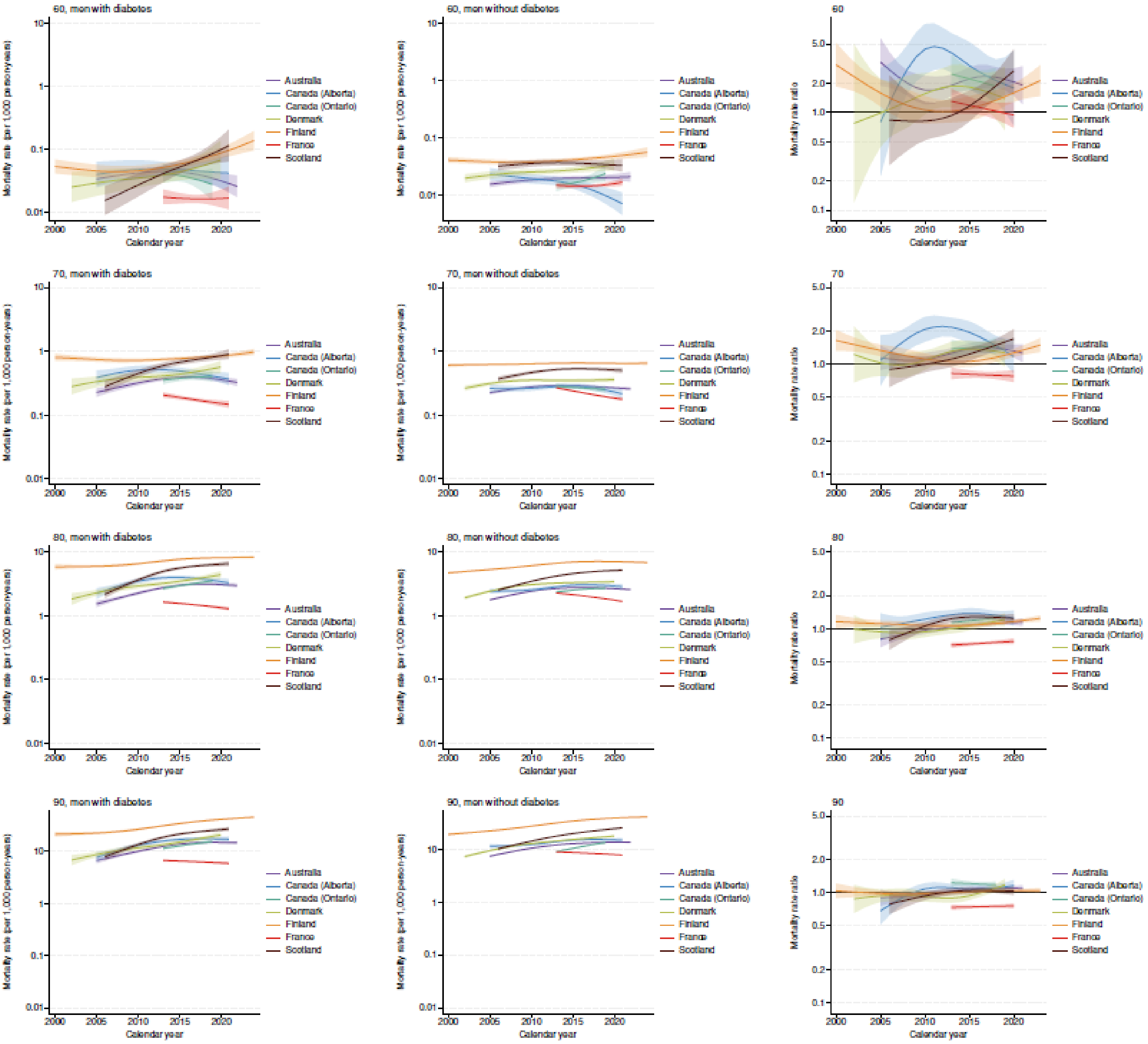

ESM Fig. 2. Dementia mortality rates for people with and without diabetes and mortality rate ratios for people with diabetes vs. those without diabetes by calendar time at 60, 70, 80 and 90 years of age, men. Data are expressed as mean (line) and 95% CI (shaded area)

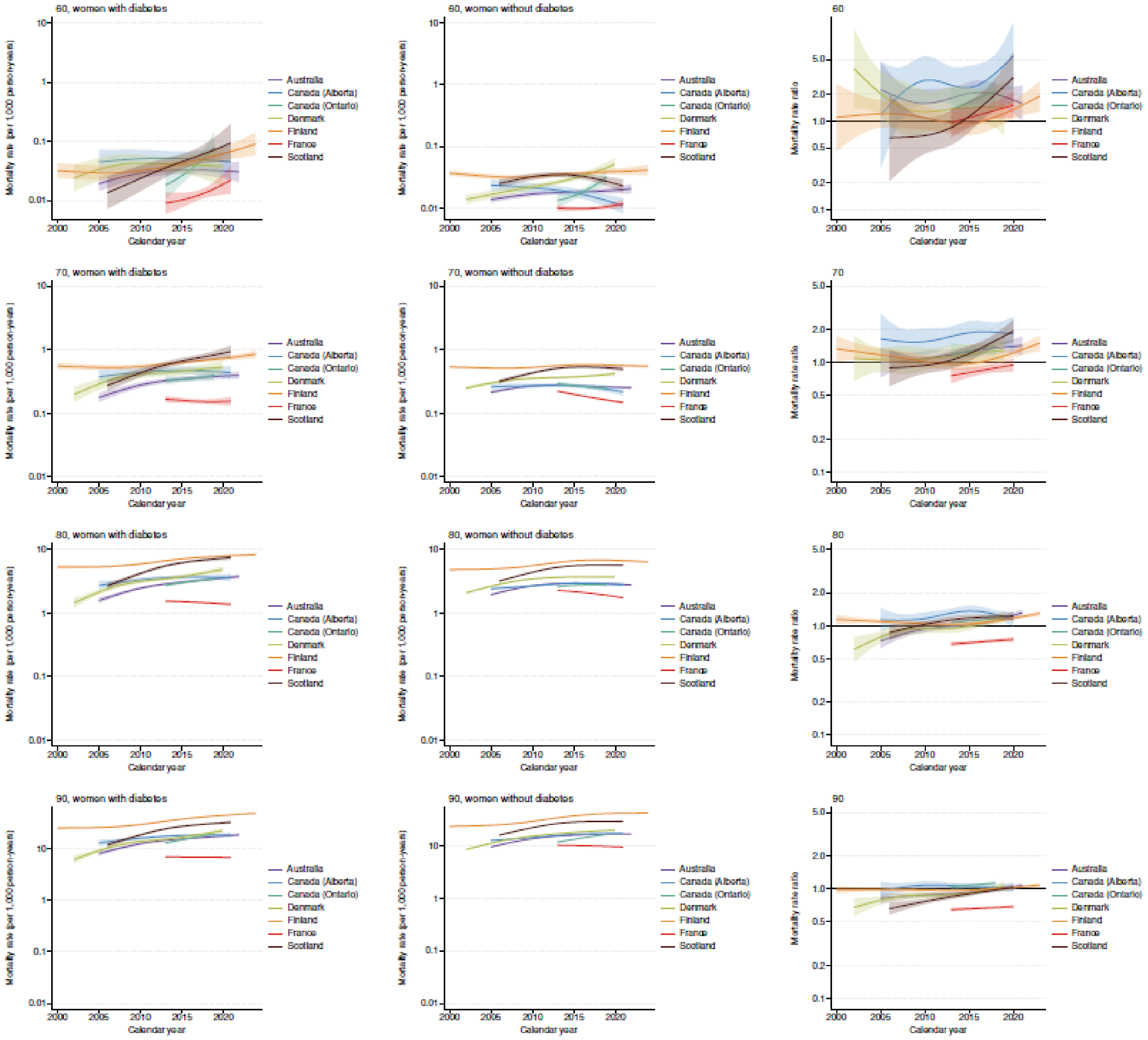

ESM Fig. 3. Dementia mortality rates for people with and without diabetes and mortality rate ratios for people with diabetes vs. those without diabetes by calendar time at 60, 70, 80 and 90 years of age, women. Data are expressed as mean (line) and 95% CI (shaded area)

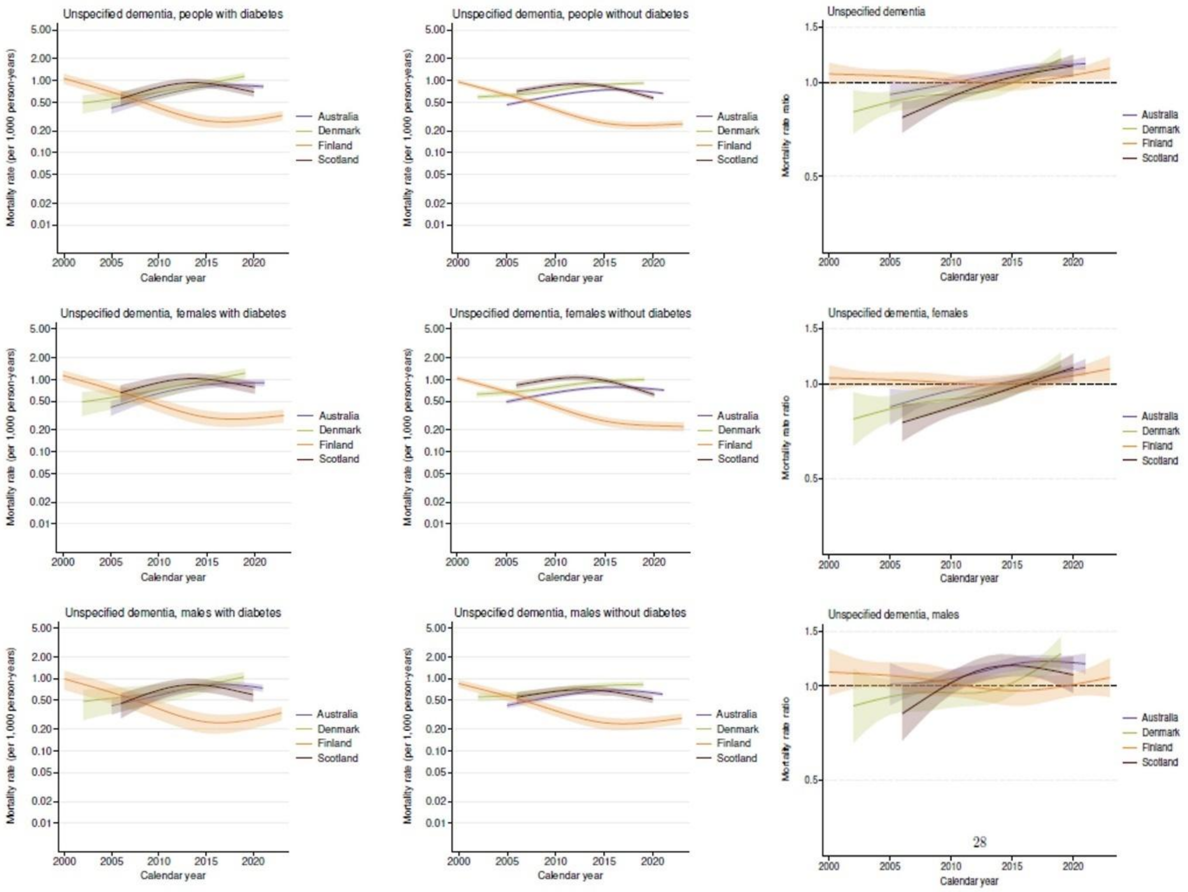

ESM Fig. 4. Age-standardised unspecified dementia mortality rates for people with and without diabetes and mortality rate ratios for people with diabetes vs. those without diabetes by calendar time, people aged 40–89. Data are expressed as mean (line) and 95% CI (shaded area)
